# Cost of Public Health Protection

**Published:** 1915-06

**Authors:** 


					﻿Cost of Public Health Protection. Public Health, February,
1915. (Note: The charge against the District of Columbia
is largely national.)
34___________________________________________  34
33	   33
«______________________________________________32
2	31_______________________________________________31
5
*8	30______________________.__________________________ 30
“	29	29
g	28 _____________________________________________28
g	27______________________________________________27
o
|	26______________________________________________26
|	25--------------------------------------------  25
I	24----------------------------------------------24
3	23----------------------------------------------23
§
a.	22______________________________________________22
«
°	21_______________________________________________21
1“	20______________________________________________20
m	19----------------------------------------------19
§
g	18----------------------------------------------18
|	17______________________________________________17
x	16----------------------------------------------16
§	15______________________________________________15
3	14______________________________________________14
K
§	13______________________________________________13
5
12 — ___________________________________________12
11______________________________________________11
U
g	10______________________________________________10
?	9--------------------------------------------  9
§	«______________________________________________.
85
~	7_____________________________________________   7
0
g	6------------------------------------------------6
I	•—--rm-------------------------------------------•
g	4________________________________________________4
§	3-------------. ..	_________________________ 3
n,
° 2___________________________ ___________ ____________ 2
J :~rnrlll 1111111 Illi IIII Illi ii ii ii i
F»	i H I I I ! * * *........* ••>••• ...........
• ••ZZZ11Z..................................
O	..••••••••••••••••••••••♦•...................
2;	• •;!!!!!........................••••••••••
t • Z Z Z...........••••••••••••••••••••
• • • • • ......................• •••••••
x	e l ! 1	...............♦ •••••••........•
3»ZZZZZZ.....................................
x	o Z Z Z Z Z Z................................
X	r. -
' S	: : § i : : j?’ J U1i J H j J : : ’• H :
b £ f; r C	~	i	w T’ *5 c	o •	5 R •- T< rj
w£t>cEfc'S'££^r'>S°. 81	$*>«8£'dcu)NOK-p(Q»}^1c
				

